# The relationship between teamwork, moral sensitivity, and missed nursing care in intensive care unit nurses

**DOI:** 10.1186/s12912-023-01400-y

**Published:** 2023-07-26

**Authors:** Monir Nobahar, Malihe Ameri, Shahrbanoo Goli

**Affiliations:** 1 Nursing Care Research Center, Semnan University of Medical Sciences, Semnan, Iran; 2 Student Research Committee, Semnan University of Medical Sciences, Semnan, Iran; 3 Department of Biostatistics, Health Related Social and Behavioral Sciences Research Center, Shahroud University of Medical Sciences, Shahroud, Iran

**Keywords:** Teamwork, Moral sensitivity, Missed nursing care, Intensive care unit, Nursing

## Abstract

**Background:**

Teamwork, moral sensitivity, and missed nursing care are important healthcare challenges for Intensive Care Unit (ICU) nurses and the existence of a relationship between these variables can be useful for developing better care improvement strategies. This study aimed to determine the relationship between teamwork, moral sensitivity, and missed nursing care in ICU nurses.

**Methods:**

This is a descriptive cross-sectional study conducted on a total of 200 ICU nurses working at teaching hospitals affiliated to Semnan and Shahroud Universities of Medical Sciences, Semnan, Iran in 2022. Sampling was conducted using the census method. Data collection was conducted using a demographic checklist, the TeamSTEPPS Team Perception Questionnaire (T-TPQ), Lützén Moral Sensitivity Questionnaire (L-MSQ), and Kalisch and Williams Missed Nursing Care (MISSCARE) Survey. The examination of the relationship between the three variables was conducted using Pearson’s correlation coefficient and multiple regression analysis.

**Results:**

The mean and standard deviation of teamwork, moral sensitivity, and missed nursing care was 3.47 ± 0.69, 64.19 ± 13.43, and 55.04 ± 34.10, respectively. The variable of teamwork had a significant positive relationship with moral sensitivity (*p* < .001) and a significant negative relationship with missed nursing care (*p* < .001). Teamwork was also a positive predictor of moral sensitivity (*p* < .001) and a negative predictor of missed nursing care (*p* < .001). The clinical experience of ICU nurses was a positive predictor of teamwork (*p* = .01) and a negative predictor of missed nursing care (*p* = .001). The age of ICU nurses was a positive predictor of moral sensitivity (*p* = .001) and a negative predictor of missed nursing care (*p* = .008).

**Conclusion:**

The findings showed that a higher level of teamwork was associated with increased moral sensitivity and reduced missed nursing care among ICU nurses. Therefore, focusing on planning interventions on teamwork improvement can lead ICU nurses to improve moral sensitivity, lower missed nursing care, and promote the quality of patient care.

## Background

Teamwork is a process in which team members interact with each other and combine available resources to perform assigned tasks [1]. Effective teamwork is necessary for conducting safe and high-quality patient care so that it not only leads to higher patient satisfaction but also creates a healthier and happier work environment for nurses, resulting in increased job satisfaction [2]. Nurses are one of the main members of the healthcare team [3] and their teamwork plays a crucial role in promoting the quality of patient care [4, 5] especially in Intensive Care Units (ICUs) [6]. ICU nurses are responsible for providing necessary treatment and health care, and teamwork among them is a critical component of care quality [7].

Teamwork in ICU is usually occurs in clinical situations that involve ethical challenges [8, 9]. In such situations, having moral sensitivity is one of the important aspects of providing quality nursing [10]. Based on the viewpoint of Lützén et al. (2006), moral sensitivity means understanding the patient’s vulnerability and being aware of the importance of the ethical consequences of making decision in clinical situations [11]. Nurses should have decent levels of moral sensitivity because it helps them solve clinical problems [12] and effectively improve the quality of nursing care [13]. The question raised here is whether teamwork between nurses is associated with moral sensitivity. Despite a search conducted in the available databases, no clear and precise answer was found for the above question.

Apart from moral sensitivity, paying attention to the relationship between teamwork and other clinical challenges experienced by ICU nurses when providing patient care is considerably important. Since ICU nurses have to deal with multiple and sometimes complicated tasks, missed nursing care is more likely to occur in these units [14]. The concept of missed nursing care was first proposed by Kalisch (2009) and it refers to the required nursing care that has been delayed or not performed at all [15]. Missed nursing care can also lead to adverse events, decrease the quality of patient care, and increase mortality and morbidity rates [16]. Teamwork and the creation of a unified force among nurses can both reduce missed nursing care, increase patient care quality, and finally lead to patient, family, and nurse satisfaction [17]. Based on a literature review, there are limited studies on the role of teamwork in reducing missed nursing care, especially in the ICU where patients have unique care needs compared to other departments [6]. In addition to what was stated about teamwork and its relationships with moral sensitivity and missed nursing care, there is also a hypothesis representing that moral sensitivity is related to the reduction of missed nursing care. When individual values are prioritized over collective and correct values, the level of teamwork may be reduced and this can lead to an increase in missed nursing care [18]. Few studies have examined the relationship between missed nursing care and moral sensitivity [19], which can make our study as the starting point for revealing the exact relationship between them.

In the case where there is a relationship between teamwork, moral sensitivity, and missed nursing care among ICU nurses, emphasizing the importance of planning to improve teamwork can lead health systems to achieving bigger goals in patient care provision. Therefore, this study aimed to determine the relationship between teamwork, moral sensitivity, and missed nursing care in ICU nurses.

## Methods

### Design

This is a multicenter, descriptive, analytical, cross-sectional study conducted from June to October 2022 in three teaching hospitals affiliated to Semnan and Shahroud Universities of Medical Sciences, Semnan, Iran.

### Participants

ICU nurses constituted the participants in this study. Sampling was conducted using the census method. The inclusion criteria included the followings: (a) willingness to participate in the study, (b) having at least a bachelor’s degree in nursing, (c) having at least one year of work experience in the ICU, and (d) working in a single medical center. The exclusion criteria were made up of the followings: (a) incomplete questionnaires and (b) having the experience of participation in teamwork-related courses.

### Instruments

Data were collected using a demographic checklist, the TeamSTEPPS Team Perception Questionnaire (T-TPQ), the Lützén Moral Sensitivity Questionnaire (L-MSQ), and the Kalisch and Williams Missed Nursing Care (MISSCARE) Survey.

### Demographic checklist

The demographic checklist included items on age, gender, marital status, level of education, job position, and clinical work experience.

### TeamSTEPPS team perception questionnaire (T-TPQ)

The T-TPQ is an evidence-based tool to measure an individual’s attitude and the perception of teamwork knowledge, skills, and behaviors. This questionnaire includes 35 items in five subscales, including “team structure”, “team leadership”, “situational monitoring”, “mutual support” and “communication”. Each subscale consists of seven items, all of which are scored on a 5-point Likert scale from “Strongly disagree = 1” to “Strongly agree = 5”. A mean score of more than 3 indicates acceptable teamwork. Cronbach’s alpha coefficient of the Persian version of this questionnaire was shown to be 0.96 [20].

### Lützén moral sensitivity questionnaire (L-MSQ)

The L-MSQ is a 25-item tool developed by Lutzen et al. (2006) [11] and adjusted by Comrie (2012). This questionnaire contains six dimensions, including “respect for the client’s independence”, " knowledge of how to communicate with the patient “, “professional knowledge”, “experience of problems and ethical conflicts”, “tapplication of moral concepts in moral decision”, and “honesty and benevolence”. All items of this questionnaire are scored based on a 5-point Likert scale from “Neutral = 0” to “Strongly agree = 4”. The overall score of this tool ranges from 0 to 100, based on which moral sensitivity is classified into three levels of low (0–50), medium (50–75), and high (75–100) [21]. Cronbach’s alpha coefficient of the Persian version of this questionnaire was obtained to be 0.81 [12].

### Kalisch and Williams missed nursing care (MISSCARE) survey

The MISSCARE Survey is a 24-item tool developed and psychometrically assessed by Kalisch and Williams (2009) and each of its items is about a particular part of nursing care [22]. This questionnaire is made up of four subscales, including “care intervention with ongoing assessment”, “intervention for individual needs”, “basic care intervention” and “discharge planning and patient education”. All items of this survey are scored on a 5-point Likert scale from “Never = 0” to “Always = 4”. The overall score of this survey fluctuates between 24 and 96, so that the higher the score is, the more the nursing care is missed. The score of missed nursing care can be converted into a percentage-based result. Based on the overall score, missed nursing care is classified into three levels of low (less than 60%), medium (60–75%), and high (more than 75%) [23]. Cronbach’s alpha coefficient of the Persian version of this questionnaire was calculated to be 0.91 [24].

### Procedure

Prior to the data collection, the necessary approvals were obtained from the Vice-Chancellor for Research of Semnan and Shahroud Universities of Medical Sciences. To collect data, electronic questionnaires were created using Google Forms. Online data collection enabled us to recruit ICU nurses from several medical centers. The participants had no need for a Google account to complete the four-section survey. It was possible to move forward and backward using the “Next” and “Back” buttons. The questions were not required to be mandatorily answered. The researcher held face-to-face meetings and phone calls with ICU head nurses to provide them with information about the study, including the type of study, objectives, eligibility criteria, and participation process. The head nurses assisted us to provide the questionnaires link for the eligible nurses, who completed and submitted the questionnaires within a week as planned.

### Data analysis

All processes of data analysis were conducted using IBM SPSS Statistics for Windows, version 20 (IBM Corp., Armonk, N.Y., USA). Data were presented using descriptive statistics, including frequency, percentage, mean, and standard deviation. Pearson’s correlation coefficient was utilized to examine the relationships between the three main variables. The examination of the predicting role of teamwork, moral sensitivity and missed nursing care was conducted using multiple regression analysis. A *p*-value of less than 0.05 was considered significant for all statistical tests.

## Results

### Demographic information of the participants

The rate of response to the questionnaire was 74.82%. A number of 14 incomplete questionnaires excluded and a total 200 questionnaires were finally included in the data analysis process. Based on the data obtained, 79% of participants were female and their mean (± Standard Deviation (SD)) age was 32.7 ± 5.65 years (Table 1).

**Table 1:** Characteristics of ICU nurses

Variables		M ± SD	n (%)
Age (year)		32.7 ± 5.65	-
Gender	Male	-	42 (21)
	Female	-	158 (79)
Marital status	Married	-	110 (55)
	Unmarried	-	90 (45)
Education level	Bachelor	-	169 (84.5)
	Master or PhD	-	31(15.5)
Work positon	Staff	-	152 (76)
	Charge	-	48 (24)
Clinical experience (year)		6.87 ± 2.26	-

M = mean; SD = standard deviation

### Outcome

The mean score (± SD) of teamwork was 3.47 (± 0.69) and this showed an acceptable level of teamwork. The standard mean was used to compare the subscales of teamwork. The highest mean scores (± SD) belonged to the subscales of “mutual support” (90.29 ± 17.69) and “team structure” (89.55 ± 16.52) and the lowest one was shown to be for the subscale of “situational monitoring” (82.62 ± 18.82).

The mean score (± SD) of moral sensitivity was 64.19 (± 13.43) indicating its medium level among participants. Based on the comparisons made between the dimensions of moral sensitivity, the highest mean score (± SD) was obtained for two dimensions of “knowledge of how to communicate with the patient” (73.07 ± 19.02) and “the experience of problems and ethical conflicts” (67.63 ± 17.35), while the lowest one belonged to the dimension of “professional knowledge” (53.64 ± 21.22).

The mean score (± SD) of missed nursing care was 34.55 (± 10.04) and the results showed that the highest mean score of missed nursing care was for the dimension of “discharge planning and patient education” (60.16 ± 20.78), and the lowest one was for the dimension of “care intervention with ongoing assessment” (42.68 ± 13.71) (Table 2).

**Table 2:** Overal index according to team work, moral sensitivity and missed care dimentions

Variables	Domain	Item no	M ± SD
Teamwork	Team structure	7	89.55 ± 16.52
	Team leadership	7	83.20 ± 21.71
	Situational monitoring	7	82.62 ± 18.82
	Mutual support	7	90.29 ± 17.69
	Communication	7	88.69 ± 20.80
Moral sensitivity	Respect for the client independence	3	64.50 ± 16.52
	Knowledge of how to communicate with the patient	5	73.07 ± 19.02
	Profetional knowledge	2	53.64 ± 21.22
	The experience of problems and ethical conflicts	3	67.63 ± 17.35
	The application of moral concepts in moral decision	5	64.05 ± 16.99
	Honesty ane benevolence	7	59.00 ± 16.60
Missed care	Care intervention with ongoing assessment	8	42.68 ± 13.71
	Intervention for indivisual needs	6	46.46 ± 15.26
	Basic care intervention	7	50.47 ± 16.98
	Discharge planning and patient education	3	60.16 ± 20.78

M = mean; SD = standard deviation

### Correlation between variables

The findings of this study also revealed that teamwork had a significant and positive relationship with moral sensitivity (*r* = .48) and a significant and negative relationship with missed nursing care (*r* = − .44). There was also a significant and negative relationship between moral sensitivity and missed nursing care (*r* = − .36) (Table 3).

**Table 3:** Correlation of team work, moral sensitivity and missed care

Variabeles	Teamwork	Moral sensitivity
	r (P)	r (P)
Teamwork	-	0.48 (< 0.001)
Moral sensitivity	0.48 (< 0.001)	-
Missed care	-0.44 (< 0.001)	-0.36 (< 0.001)

M = mean; SD = standard deviation; r = Pearson correlation

A statistically significant relationship at P < .05 Pearson’s coefficient

The results of multiple regression analysis indicated that teamwork was a positive predictor of moral sensitivity (*p* < .001) and a negative predictor of missed nursing care (*p* < .001). Furthermore, the clinical experience of ICU nurses was a positive predictor of teamwork (*p* = .01) and a negative predictor of missed nursing care (*p* = .001). It was also found that the age of ICU nurses was a positive predictor of moral sensitivity (*p* = .001) and a negative predictor of missed nursing care (*p* = .008) (Table 4).

## Discussion

In this study, ICU nurses were shown to have acceptable, moderate, and low levels of teamwork understanding, moral sensitivity, and missed nursing care, respectively. Moreover, teamwork was shown to be a predictor of moral sensitivity and missed nursing care. It is noteworthy that the mean scores of teamwork in most of the studies conducted in recent years are higher than in the past [25–27]. This discrepancy between the findings can be due to higher attention paid to the improvement of care quality and organizational culture affecting nurses’ understanding of teamwork.

The results of this study revealed that “mutual support” was the most important dimension of teamwork. There was no study with consistent results in this area. The results of a study by Çeli̇k et al. (2019) indicated that this dimension had the lowest mean score [28]. Deeper studies can help us better understand the causes of these differences. Furthermore, the results of this study showedthat “team structure” also obtained a high score among the dimensions of teamwork. Nurses are strongly recommended to have structured cooperation to achieve effective teamwork [4]. The results of our study also demonstrated that the lowest score among the areas of teamwork belonged to the “situational monitoring”. This dimension obtained a high score compared to other areas in studies conducted by Weaver et al. (2017) [29] and Hall-Lord (2020) [30]. Despite that the above studies were conducted in different working environments, this does not seem to be the reason for this discrepancy in results. It should be noted that the results of this particular part of the similar studies conducted in Iran are in line with the results of our study, although they were all conducted in different working environments [4, 31]. The reason for this discrepancy in the results might be the cultural differences between nurses in different countries.

Based on the results of our study, it was also found that moral sensitivity was at a moderate level among ICU nurses. This finding is in line with the results of studies conducted by researchers in Iran [12, 13] and Ye et al. (2022) in China [32]. This result shows that despite facing many complex clinical situations, ICU nurses do not have decent and necessary levels of moral sensitivity to face such situations yet [33]. Accordingly, it is necessary to pay more attention to moral sensitivity among health professionals working in ICUs.

**Table 4:** Factors predicting ICU nurses teamwok, moral sensitivity and missed care

Variabeles	Teamwok**			Moral sensitivity***			Missed care****		
	β	P	CI	β	P	CI	β	P	CI
Age	-0.12	0.26	-1.49–0.41	0.33	0.001*	0.31–1.23	-0.27	0.008*	-1.23 - -0.18
Gender	0.009	0.89	-7.80–8.92	0.02	0.66	-3.20-4.99	-0.10	0.10	-8.21–0.77
Degree	0.35	0.64	-13.93–8.63	0.02	0.71	-0.73-1.01	0.04	0.54	-0.67- 1.26
Work positon	0.08	0.30	-4.17-13.18	0.06	0.33	-2.24-6.54	-0.08	0.27	-7.62–2.19
Clinical experience	0.31	0.01*	0.32–2.51	-0.20	0.053	-1.04- 0.11	-0.35	0.001*	0.37–1.53
Teamwok	-	-	-	0.47	< 0.001*	0.19–0.33	-0.47	< 0.001*	-0.36 - -0.20

*A statistically significant relationship at P < .05 level

**Adjusted R = .05, F = 3.06, P = .01

*** Adjusted R = .27, F = 12.40, P = < 0.001

****Adjusted R = .22, F = 10.09, P = < 0.001

We also came to the result that the moral sensitivity of ICU nurses to the dimension of " knowledge of how to communicate with the patient " was more than other dimensions, which is in line with the results of most studies conducted in recent years [13, 34, 35]. ICU nurses who have the necessary knowledge to work in a complicated clinical-communicational setting [5] can help create better communication with patients in such situations. In addition, the moral sensitivity of ICU nurses to the dimension of “the experience of problems and ethical conflicts” was significantly high, which is consistent with the results of some studies [38] and inconsistent with the results of some other studies [34]. The experience of ethical conflicts can indicate the ability of nurses to identify clinical situations with moral dilemmas. So some measures should be taken to deal with and control the tension created by such situations [13]. The findings also revealed that the moral sensitivity of ICU nurses to the dimension of “professional knowledge” is lower than other dimensions. Considering the necessity and importance of promoting the level of professional knowledge among ICU nurses, nursing managers should design and execute exact plans in this regard.

It was also pointed out that missed nursing care was at a low level in our study and this result is in line with the results of the majority of recent studies [24, 36, 37]. Moreover, nursing care was mainly missed in the dimension of “discharge planning and patient education”. Patient education and discharge planning are integral parts of the rehabilitation of patients with complicated health conditions [38]. It seems that studies that deeply investigate this issue will help to identify the reasons for the loss of this important part of the care provided by ICU nurses. Nursing care was indicated to be minimally missed in the dimension of “care intervention with ongoing assessment”, which includes services such as monitoring vital signs, blood sugar, intake, and output. This finding is consistent with the results of other related studies [18, 22, 36, 39] and it seems that the care services that require form recording are less missed than other ones. Accordingly, it is necessary to pay more attention to the necessity of using monitoring forms and up-to-date documentation systems in nursing care.

The results of this study revealed that ICU nurses’ clinical experience was a positive predictor of teamwork. Welp et al. (2019) concluded that the members of the health care team who had higher clinical work experience in the ICU were more capable of conducting teamwork affairs [40]. Caring for a patient with complicated critical care needs requires stronger teamwork [5] and it seems that ICU nurses can gain a better understanding of the necessity of teamwork and achieve nursing care goals over time by increasing their clinical experience. The results also led us to this.

conclusion that increased clinical experience was a negative predictor of missed nursing care and this conclusion is in line with the findings of the study conducted by Soliman and Eldeep (2020) [14]. However, Ghezeljeh et al. (2021) found that there is no significant relationship between these two variables [41]. The reason for this inconsistency between the results may be due to the difference in patients receiving care and the clinical environments. So it is suggested to conduct more studies in this area, especially in ICUs.

Based upon the findings, it was shown that the age of ICU nurses was a positive predictor of moral sensitivity. Borhani et al. (2016) also reached similar results in this area, but Amiri et al. (2019) pointed out that there was a reverse relationship between age and moral sensitivity [13]. Due to the existence of conflicting results in studies conducted in this field, determining the role of increasing age in improving nurses’ moral sensitivity will require further research. We also found that the age of nurses was a negative predictor of missed nursing care. In other words, the probability of missing nursing care decreased as the age went up. Some studies had the same findings in this part [39, 42].

The results showed that there was a significant relationship between the three main components of the study, including teamwork, moral sensitivity, and missed nursing care. The higher levels of teamwork improved moral sensitivity and decreased the number of missed nursing care. Moreover, as the major point in the results of this study, teamwork was a positive predictor of moral sensitivity and a negative predictor of missed nursing care. Based on the literature review, there was no study that examined the relationship between these three components simultaneously. However, there were few studies that merely examined the relationship between teamwork and missed nursing care [41], the results of which were in line with the findings of our study. The result of this study provides more evidence about the need for teamwork to improve patient care. Teamwork and moral sensitivity are one of the most important concerns of nurse researchers and there have been several interventions designed to improve ICU nurses’ performance in this regard [43]. Evidence-based nursing has been always looking for interventions that, in addition to being effective, can be implemented [44]. In this study, teamwork was correlated with important moral and care-associated challenges in the ICU, and the concentration of interventions on it can help achieve useful results in solving the mentioned challenges. We also came to the result that moral sensitivity and missed nursing care had a significant and negative relationship. Due to the fact that few studies have been conducted on the relationship between the above variables so far [19], it is not possible to compare the results. However, the results of a study by Vryonides et al. (2018) showed that if nurses feel that the moral atmosphere governs their work environment, the occurrence probability of missed nursing care decreases [18]. Therefore, this results can be considered a beginning point for conducting more comprehensive studies. Despite that data were collected from different centers, there were also limitations in this study that limit the generalizability of the results. First, Cross-sectional methods can limit the evaluation of the relationships between different variables in the subsequent research and it should be noted that causal interpretation is not possible. In addition, regarding that the data collection tools were all self-report, the occurrence of bias was possible. Therefore, the results should be applied with more caution.

## Conclusion

It was concluded that nurses with a higher understanding of teamwork had higher levels of moral sensitivity and a lower rate of missed care. It was also concluded that there was a significant and negative relationship between moral sensitivity and missed nursing care. Teamwork can have valuable implications for ICU nurses when making ethical decisions and providing patient care. Getting through this way can lead healthcare systems to create favorable results in the provision of quality patient care by setting multiple goals and a single joint intervention.

## Data Availability

The datasets generated and analyzed during the current study are not publicly available due to privacy protection and ethical considerations but are available from the corresponding author upon reasonable request.

## Abbreviations

ICU: Intensive Care Unit
T-TPQ: TeamSTEPPS Team Perception Questionnaire
L-MSQ: Lützén Moral Sensitivity Questionnaire
MISSCARE: Missed Nursing Care
SD: Standard Deviation
